# Adaptive capacity of 2- to 5-month-old infants to the flow, shape, and flexibility of different teats during bottle feeding: a cross-sectional study

**DOI:** 10.1186/s12887-019-1859-y

**Published:** 2019-12-05

**Authors:** M. L. J. Lagarde, N. van Alfen, S. A. F. de Groot, A. C. H. Geurts, L. van den Engel-Hoek

**Affiliations:** 1 Radboud University Medical Center, Department of Rehabilitation, Donders Institute for Brain, Cognition and Behavior, Geert Grooteplein 10, 6525 GA Nijmegen, the Netherlands; 2 Radboud University Medical Center, Department of Neurology, Donders Institute for Brain, Cognition and Behavior, Nijmegen, the Netherlands

**Keywords:** Nutritive sucking, Bottle feeding, Swallowing, Infants, Coordination sucking-swallowing-breathing

## Abstract

**Background:**

Nutritive sucking is a complex activity, the biomechanical components of which may vary in relation to respiratory phase, swallow-rate per minute, suck-swallow ratio, and swallow non-inspiratory flow (SNIF). Quantitative measurement of these components during nutritive sucking in healthy infants could help us to understand the complex development of sucking, swallowing, and breathing. This is important because the coordination between these components is often disturbed in infants with feeding difficulties. The aims of this study were to describe the biomechanical components of sucking and swallowing in healthy 2- to 5-month-old infants during bottle feeding, to assess whether infants adapt to the characteristics of two different teats, and to determine which independent variables influence the occurrence of SNIF.

**Methods:**

Submental muscle activity, nasal airflow, and cervical auscultation were evaluated during bottle-feeding with two different teats.

**Results:**

Sixteen term-born infants (6 boys) aged 2–5 months were included. All infants showed variable inhalation and exhalation after swallowing. The swallow rate per minute was significantly higher when infants fed with a higher flow teat (Philips Avent Natural 2.0™). Infants had suck:swallow ratios ranging from 1:1 to 4:1. A suck:swallow ratio of 1:1 occurred significantly more often when infants fed with a higher flow teat, whereas a suck:swallow ratio of 2:1 occurred significantly more often when infants fed with a low-flow teat (Philips Avent Classic+™). A suck:swallow ratio of 1:1 was negatively correlated with SNIF, whereas a suck:swallow ratio of 2:1 was positively correlated with SNIF.

**Conclusion:**

Healthy infants aged 2–5 months can adapt to the flow, shape, and flexibility of different teats, showing a wide range of biomechanical and motor adaptations.

## Background

Adequate nutritional intake during breast or bottle feeding is essential for the proper growth and development of neonates. Nutritive sucking is a complex activity consisting of well-coordinated sucking, swallowing, and breathing [1]. Problems during nutritive sucking may lead to oxygen desaturation, bradycardia, and aspiration of liquid into the lungs, which can cause pneumonia and a dependence on tube feeding [2].

Nutritive sucking involves a number of biomechanical aspects, namely, (i) swallowing in relation to respiratory phase, (ii) the suck:swallow ratio, and (iii) swallowing rate per minute. The coordination between swallowing and respiration changes with age [1, 3, 4]. In both preterm and term infants, swallowing can be followed by both inhalation and exhalation, but the frequency of exhalation after swallowing increases during the first year of life [4–7]. Shortly after birth, infants drink with a suck:swallow ratio of 1:1, but after 1 month the suck:swallow ratio is higher, 2:1 [1]. The swallowing rate per minute is dependent on the suck:swallow ratio, and a swallowing rate of 60/min has been described in preterm and newborn infants [6]. Less information is available on the biomechanical aspects of normal nutritive sucking in healthy, term infants after the age of 2 months, when feeding reflexes have disappeared. It is assumed that biomechanical aspects are different in infants older than 2–3 months than in younger infants [8]. Quantitative measurement of these biomechanical aspects in healthy infants during nutritive sucking might help us to understand the complex development of sucking, swallowing, and breathing, especially because the coordination between these aspects is often disturbed in infants with feeding difficulties. Adjusting teats is one of the possible interventions in the management of infants with feeding difficulties. Based on the knowledge of adaptation in normal motor development [9] it is hypothesized that healthy infants are able to adapt to flow and shape of a teat. The information about healthy infants is needed to support decision making in the management of infants with feeding difficulties.

Another aspect of swallowing is the phenomenon of swallow non-inspiratory flow (SNIF). Directly after swallowing, there is a brief period of non-respiratory airflow in adults [10–12]. SNIF occurs when the laryngeal vestibule opens and the tongue base and soft palate are released from the pharyngeal wall [10]. This inward airflow releases the vacuum that develops at the end of pharyngeal muscle contraction [10]. Little is known about the relationship between SNIF and the phase of respiration and swallowing (inhalation or exhalation after swallowing). SNIF occurs less often in older adults (above 80 years) than in younger healthy persons [10]. Although SNIF has been described in premature infants during non-feeding swallowing (saliva), it has not been described during nutritive sucking [13]. Nothing is known about the relevance of SNIF during normal swallowing. Knowledge of how SNIF develops in healthy young subjects may make it possible to detect deviant patterns of SNIF in patients with swallowing problems.

Teats that mimic the shape, flexibility, and flow of the female nipple are popular. Bottled-fed newborn infants show significant differences in breathing and sucking frequencies when feeding with a high-flow versus low-flow teat [14–16]. While infants in the reflexive phase of nutritive sucking are able to adapt to teats with different flows, it is unclear whether infants are able to adapt to different teat characteristics after feeding reflexes (rooting and sucking reflex) have disappeared.

The aims of this study were threefold: [1] to describe normal biomechanical aspects of nutritive sucking during bottle feeding (coordination of breathing and swallowing, suck:swallow ratio, and occurrence of SNIF) in 2- to 5-month-old infants, [2] to assess whether healthy, term infants aged 2–5 months are able to adapt to the characteristics of a different teat (flow, shape, and flexibility) by adjusting biomechanical aspects of sucking and swallowing, and [3] to assess whether there are independent variables (suck:swallow ratio, inhalation/exhalation after swallowing) that influence the occurrence of SNIF. We hypothesized that healthy term infants are able to adapt to the flow and shape of different teats by adjusting the coordination of sucking, swallowing, and breathing.

## Methods

### Design

This cross-sectional study was carried out at the outpatient clinic for Speech and Language Pathology at the Radboud University Medical Center in Nijmegen between February 2017 and September 2017.

### Subjects

Healthy 2-to 5-month-old infants were enrolled after their parents gave written informed consent. Recruitment was via an announcement in child health centers in Nijmegen, the Netherlands. Bottle-fed infants or those that had a combination of breast feeding and bottle feeding (regardless of teat type) were included. Premature infants or infants with feeding difficulties were excluded. The age, sex, and weight of the infants were recorded. The study was approved by the regional medical ethics committee.

### Measurements

**Fig. 1:**
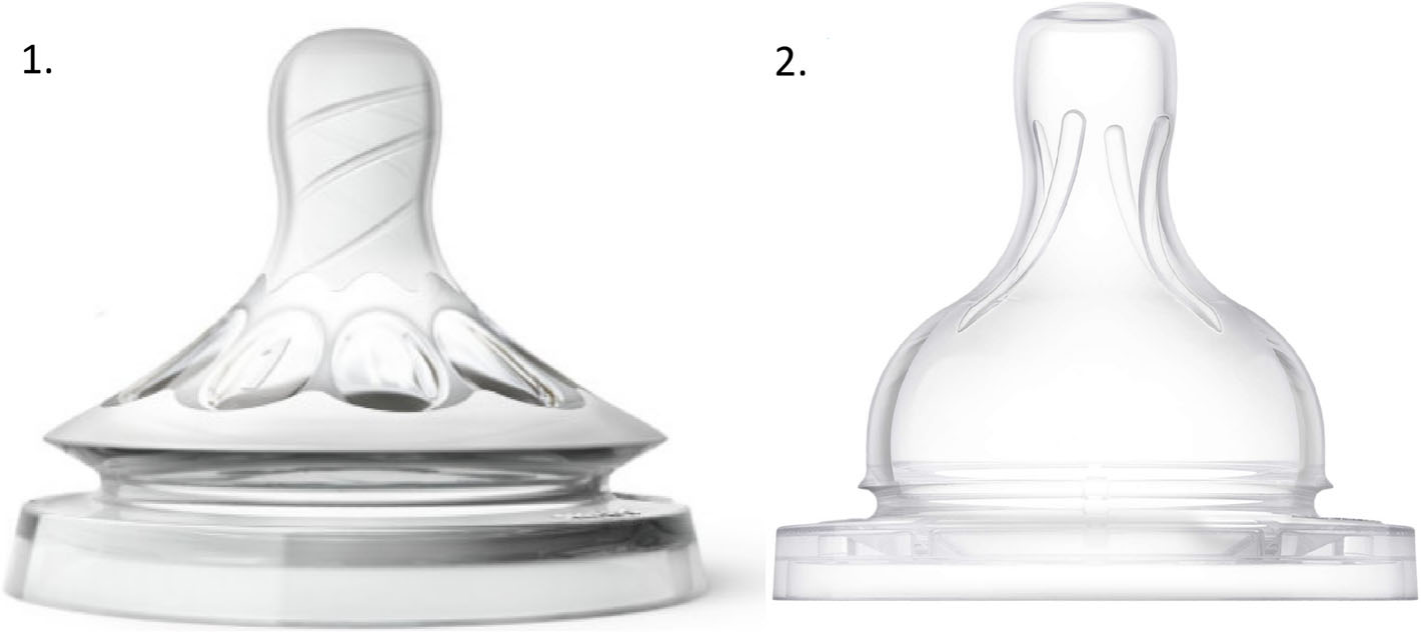
Teat 1 and teat 2 used during the measurements. 1. Philips Avent Natural 2.0 teat; 2. Philips Avent Classic+ teat

**Fig. 2:**
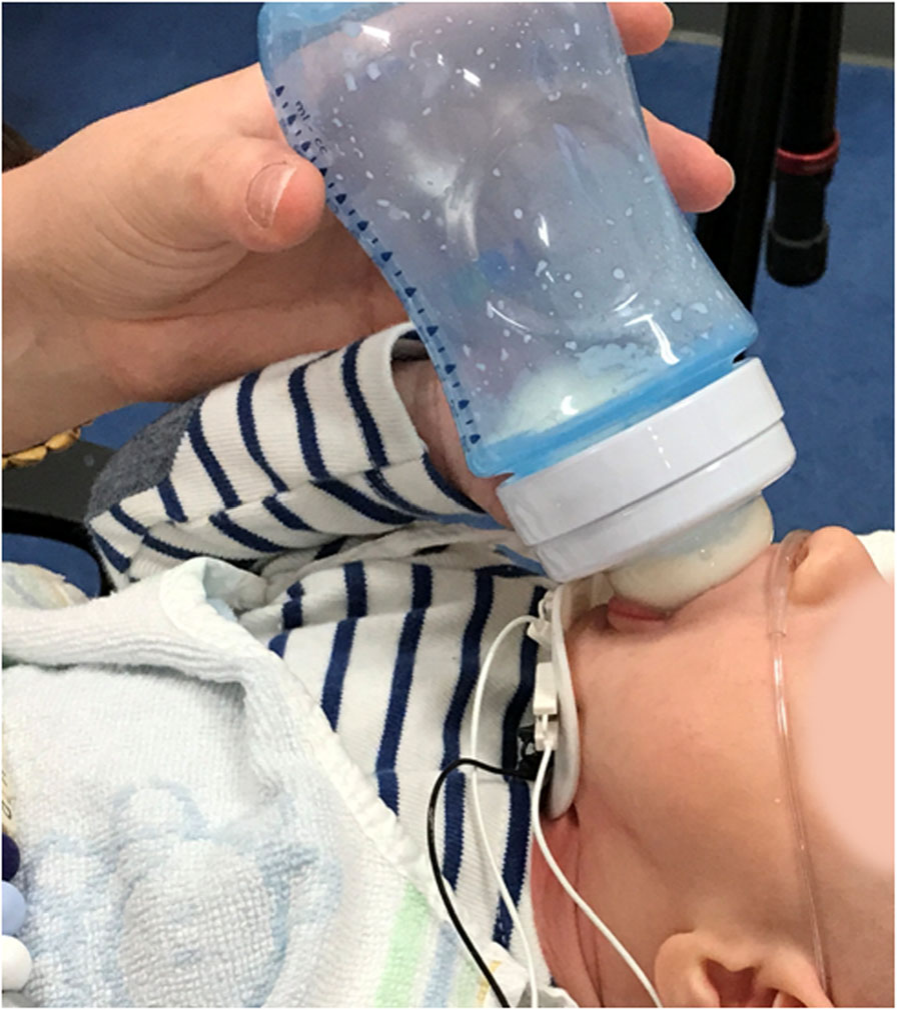
Placement of sEMG electrode and nasal cannula

All infants drank expressed breast milk or their usual formula from feeding bottles with a Philips Avent Natural™ teat 2.0(teat 1) or a Philips Avent Classic+™ teat (teat 2) and were fed by one of their parents. Both teats had flowrate 1, which is designed for babies of 0+ months old. The teats have a different shape, flow, and flexibility. Teat 1 is an extra soft, wide, breast-shaped teat, which according to the manufacturer had a higher flow than teat 2. Teat 2 is a soft wide teat, which according to the manufacturer had a lower flow than teat 1 (Fig. 1) (personal communication from Philips Avent). The exact details of the teats (flow-rate in mL/sec) were not important for this study, because we wanted to assess whether infants are able to adapt to a different teat. Data were collected during bottle feeding with both teats, using the Digital Swallowing Workstation (DSW, KayPentax, USA), to assess whether the infants showed differences between the two teats in the coordination of biomechanical aspects of nutritive sucking. Infants were in reclined position during the measurements. A combination of measurements was recorded: muscle activity using surface electromyography (sEMG), acoustic signals during swallowing, video recording of the participant, and direction of airflow, assessed by means of a nasal cannula [17]. The equipment was placed once to perform measurements for both teats. Measurements were started after 2–3 min of continuous sucking and swallowing, to allow the infants get used to the equipment. The sEMG electrode does not put pressure on the submental muscles, so that muscle mobility is not impeded. The combination of the sEMG measurement, the acoustic signal, the airflow measurement, and the video recording of the infant was used to define sucking and swallowing movements, the coordination of swallowing and breathing (inhalation or exhalation after swallowing), and the presence of SNIF during bottle feeding. Figure 2 illustrates the placement of the nasal cannula and the sEMG electrode.

**Fig. 3:**
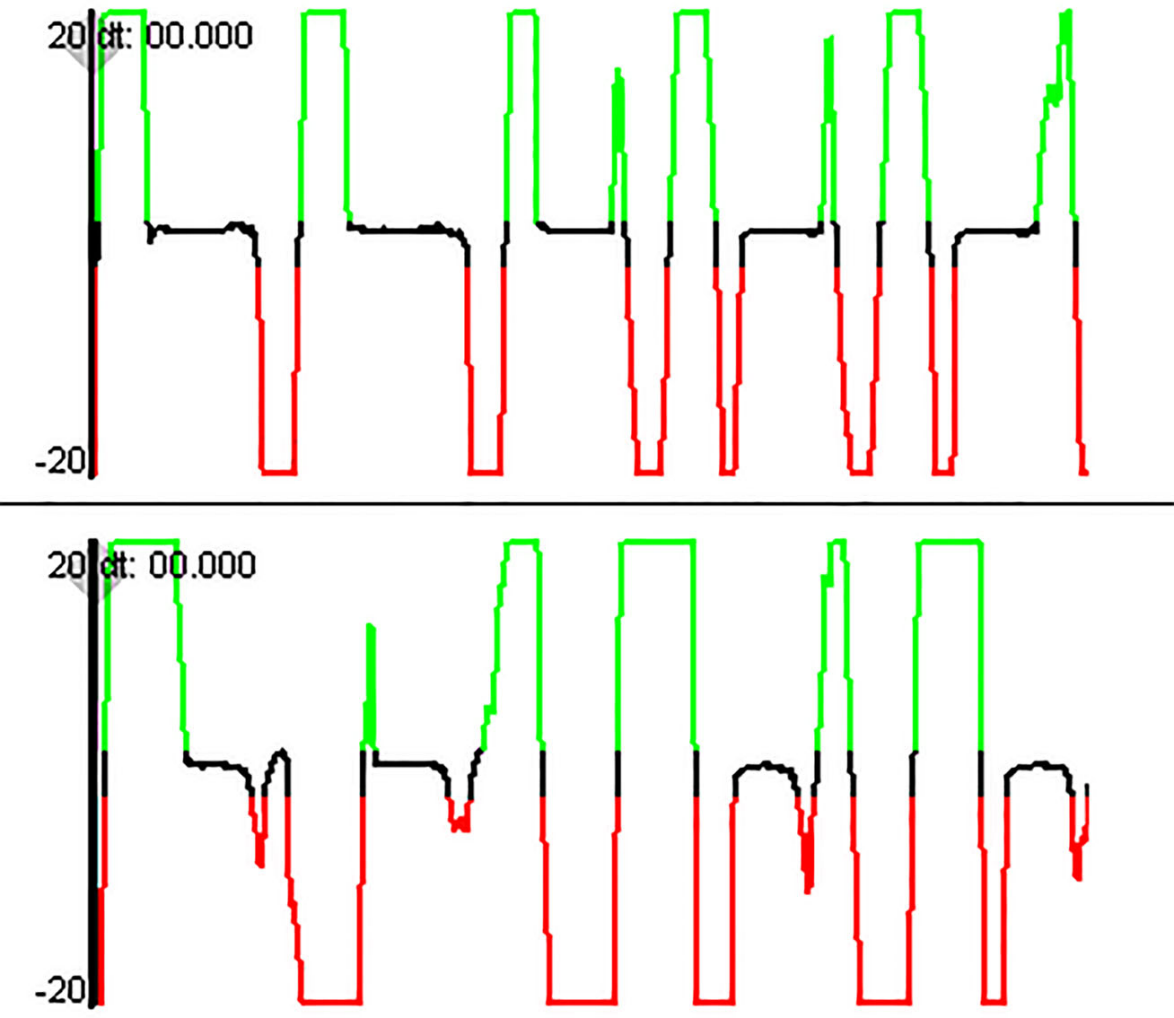
Nasal airflow measurement showing 7 s of nutritive sucking. Nasal airflow shows (**a**) no swallow non-inspiratory flow (SNIF), and (**b**) occurrence of SNIF during swallowing

For each teat, data for 1 min of feeding during the intermittent sucking phase were analyzed with the DSW. Both teats were tested in the same feeding session in a standardized order (first the Natural 2.0 teat, followed by the Classic teat). Intermittent sucking occurs after a few minutes of continuous sucking and is characterized by short bursts of sucking with pauses in between [18–20]. This phase was chosen because it was not possible to perform measurements with both teats during the short continuous sucking phase in one feeding session. Biomechanical aspects of nutritive sucking were analyzed over a 1-min period from the start of rhythmic drinking with pauses. All measurements and analyses were performed by the same investigator (ML). For analysis, the swallowing-rate (swallowing-rate per minute), the percentage of swallows followed by inhalation) and exhalation were counted. The suck:swallow ratio (number of sucking movements before swallowing) was calculated; it is described as ‘suck-swallow’ (1:1), ‘suck-suck-swallow’ (2:1), etc. The presence of SNIF during the 1-min analysis (Fig. 3) and the proportion of swallows followed by SNIF expressed as a percentage of the total swallows were determined.

### Statistical analysis

All statistical analyses were performed using IBM SPSS 22.0. Descriptive statistics were used to describe the mean and the range of the measured variables. Paired Student’s *t*-tests with a significance level of 0.05 were performed to determine whether there were significant differences in biomechanical aspects between the two teats. The correlation between the occurrence of SNIF and independent variables percentage of swallows followed by inhalation, percentage of swallows followed by exhalation, percentage of swallows with a suck:swallow ratio 1:1, 2:1, and 3:1) was plotted and calculated using Spearman’s rho. Multiple regression analysis (backward model) was used to determine which independent variables influenced the percentage SNIF.

## Results

Sixteen healthy infants (6 boys) born at term and aged 2–5 months were included (Table 1). All infants, except three, were able to feed with both teats. The three infants showed signs of stress, turned their heads away and did not start nutritive sucking when switched to teat 2. The other infants adapted to the new teat within seconds. The analyses were performed with data from the 13 infants who accepted the second teat.

**Table 1:** Characteristics of subjects

	Mean (range)/*n*
Age	3 (2–5) months
Sex	6 male / 10 female
Weight	5502 (4250–6860) grams

In total, 935 swallowing movements were analyzed. All infants showed variable inhalation and exhalation after swallowing, with exhalation after swallowing occurring more often than inhalation after swallowing regardless of the teat used, but the percentage of inhalation or exhalation after swallowing did not differ significantly between the two teats. All infants had a suck:swallow ratio of 1:1 and 2:1 at some stage during the single feeding session, although 12 infants had a suck:swallow ratio of 3:1 and 4 infants a suck:swallow ratio of 4:1. A suck:swallow ratio of 3:1 occurred during 15.2% of swallowing movements with teat 1 and 17.1% with teat 2. A suck:swallow ratio of 4:1 occurred during 2.6% of swallowing movements with teat 1 and 1.9% with teat 2. These differences were not statistically significantly. A suck:swallow ratio of 1:1 occurred significantly more often (*p* = 0.039) during nutritive sucking with teat 1 and a suck:swallow ratio of 2:1 occurred significantly more often (*p* = 0.014) with teat 2 (Table 2). The mean swallowing rate per minute was 38.0 (range 23–64) with teat 1 and 29.7 (range 19–40) with teat 2 (*p* = 0.02).

**Table 2:** Results of the measurements in 13 infants

	Teat 1 Mean (range)	Teat 2 Mean (range)	*p*-value
Swallowing-rate per minute	38.0 (23–64)	29.7 (19–40)	0.020*
Respiration after swallowing			
% Inhalation after swallowing	29.4 (9–52)	25.9 (0–79)	0.578
% Exhalation after swallowing	69.9 (48–91)	74.1 (21–100)	0.461
SNIF			
% No SNIF	15.3 (0–32)	26.3 (0–75)	0.087
% occurrence of a SNIF	84.6 (68–100)	73.7 (25–100)	0.094
Suck-swallow ratio			
% 1:1	49.3 (9–98)	33.5 (0–71)	0.039*
% 2:1	31.6 (2–62)	47.5 (14–71)	0.014*
% 3:1	15.2 (0–39)	17.1 (0–29)	0.276
% 4:1	2.6 (0–18)	1.9 (0–11)	0.594

*SNIF* swallow non-inspiratory flow

Statistically significant differences (*) between the teats, tested with paired Student’s *t*-tests

**Fig. 4:**
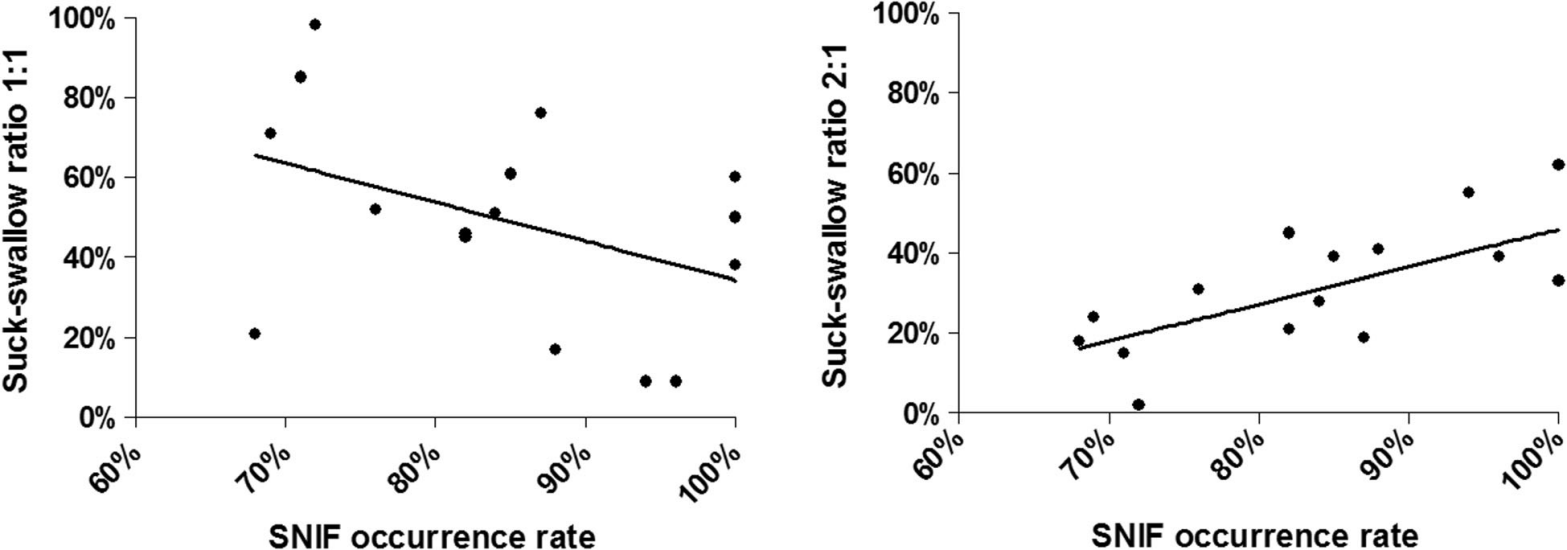
Correlations between the occurrence of swallow non-inspiratory flow (SNIF) and the suck:swallow ratio

SNIF occurred in all infants, but not after every swallow. It occurred during 68–100% of swallowing movements with teat 1 and during 25–100% of swallowing movements with teat 2. This difference was not significant. The occurrence of a SNIF (% SNIF) was significantly influenced by the suck-swallow ratio of 2:1 (%2:1) (*p* = 0.0047) explaining 46.4% of the variance. There was a negative correlation between a suck:swallow ratio of 1:1 and SNIF occurrence (*ρ* = − 0.390) but a positive correlation between a suck:swallow ratio of 2:1 (*ρ* = 0.672) and SNIF occurrence (see Fig. 4). The percentage inhalation or exhalation after swallowing did not affect the occurrence of SNIF.

## Discussion

Infants showed a broad variation in the biomechanical components of swallowing when suckling from two different teats – there were variations in swallow-rate per minute, respiration after swallowing, and suck:swallow ratio. This variation is important for normal oral motor development [9, 21, 22]. In addition, the suck:swallow ratio significantly influenced the occurrence of SNIF. For unclear reasons, three infants had difficulties nutritive sucking from teat 2 and their data were excluded from the analyses.

In our study, infants showed variable exhalation and inhalation after swallowing, as found previously in newborn infants and 1-month-old infants during bottle or breast feeding [3, 6, 7]. Kelly et al. (2007) found that the exhalation rate increased after swallowing in the first year of life [7]. In adults, exhalation after swallowing is normal [23]. The rate of exhalation after swallowing seems to increase during early infancy as a result of neurological and postnatal sensorimotor development [7, 24]. The anatomy of the oropharyngeal cavity is different in infants and adults. The small space between the soft palate and the epiglottis in infants protects the infant from inhaling liquid into the lungs [25]. This space is larger in adults, influenced by growth of the oral cavity, leading to a higher risk of aspiration when inhaling after swallowing.

The suck:swallow ratio in infants aged 2–5 months varied between 1:1 to 4:1 during one feeding session. Qureshi et al. also found a variable suck:swallow ratio in 1-month-old infants that differed from the suck:swallow ratio of 1:1 commonly seen in newborns [1]. Infants are able to collect large volumes of liquid in their mouth as a result of several sequential sucking movements and then swallow the liquid in one swallowing movement [8]. These changes probably reflect growth of the oral cavity and changes in feeding patterns as infants go from reflexive to a more volitional feeding pattern. We found differences in the swallowing rate per minute and in the suck-swallow ratio between the two teats, which indicates that healthy infants are able to adapt to different teat flow rates. Teat 1 had a higher flow rate than teat 2, which might explain the higher proportion of infants showing a suck:swallow ratio of 1:1 with this teat. Newborn infants have a varied motor repertoire and appear to be able to adapt their motor skills in response to environmental factors (differences in teat flow rate) [26, 27]. However, three infants were not able to adapt immediately to a teat with a different shape and flow. We do not know whether they would have adapted if they had been given more time. This ability to adapt to different teat characteristics is important in clinical practice, because changing teats is an intervention used to help infants with feeding difficulties. However, it also asks for adaptation of motor performance and therefore this intervention must be considered carefully and should be cue-based.

The swallowing rate per minute ranged from 29.7 to 38.0 swallows per minute. This value for 2- to 5-month-old infants is much lower than that reported by Lau et al., who reported mean swallowing rates of 45 per minute in preterm infants and 55 per minute in full-term infants aged 0–4 weeks [3]. This difference suggests that the swallow rate decreases with age as the suck-swallow ratio and volume per swallow increase with development.

SNIF occurred in all infants, but not after every swallow. Its occurrence was significantly influenced by the suck:swallow ratio. Brodsky et al. suggest that the SNIF is a result of pharyngeal pressure changes [10]. We found that SNIF occurred less often in infants with a suck:swallow ratio of 1:1 and a more frequently in infants with a suck:swallow ratio of 3:1 or 4:1. As the propulsion force exerted by the tongue seems to increase when a larger bolus is swallowed [28], the influence of the suck:swallow ratio on SNIF occurrence suggests that an increased propulsion force may lead to a need to release pressure by means of SNIF.

Our study had a number of limitations. First, we do not know the exact flow rate of the teats used and whether it was significantly different between the two teats. Previous research focused on exact flow-rate of different teats [29, 30], but the exact flow-rate of the Philips-Avent natural 2.0 teat was not previously described. Second, the teats were used in the same order in all infants, and it is possible that the infants were tired (or satiated) when drinking with the second teat, which would have altered the biomechanical properties of sucking.

Future research should focus on collecting longitudinal data on biomechanical aspects of nutritive sucking in larger groups of healthy infants and in children with feeding problems, to assess whether a limited variability in biomechanical properties is a predictor of motor development and potential feeding problems. Studies should seek to identify predictors of a decreased occurrence of SNIF, which might be related to pathology. Measurement of nasal flow during swallowing in infants with neuromuscular disorders may provide insight into the influence of muscle strength on SNIF occurrence. This information might make it possible to determine the type of intervention needed for infants with feeding difficulties.

## Conclusions

This study demonstrated that 2- to 5-month-old healthy infants show substantial variation in biomechanical aspects of nutritive sucking and can adapt to differences in the flow, shape, and flexibility of teats. In one feeding session, the suck:swallow ratio ranged from 1:1 to 4:1, depending on the flow-rate of the teat used. The swallowing rate per minute also varied among infants and between teats. Infants also showed variable inhalation and exhalation after swallowing, with exhalation after swallowing occurring more often than inhalation. The occurrence of SNIF was influenced by the suck:swallow ratio.

## Data Availability

The datasets used and/or analyzed during the current study are available from the corresponding author on reasonable request.

## Abbreviations

DSW: Digital Swallowing Workstation
sEMG: Surface Electromyography
SNIF: Swallow Non-Inspiratory Flow
