# Correction to: Adaptive capacity of 2- to 5-month-old infants to the flow, shape, and flexibility of different teats during bottle feeding: a cross-sectional study

**DOI:** 10.1186/s12887-019-1901-0

**Published:** 2020-01-30

**Authors:** M. L. J. Lagarde, N. van Alfen, S. A. F. de Groot, A. C. H. Geurts, L. van den Engel-Hoek

**Affiliations:** 1 Department of Rehabilitation, Radboud University Medical Center, Donders Institute for Brain, Cognition and Behavior, Geert Grooteplein 10, 6525 GA Nijmegen, the Netherlands; 2 Department of Neurology, Radboud University Medical Center, Donders Institute for Brain, Cognition and Behavior, Nijmegen, the Netherlands

**Correction to: BMC Pediatrics (2019) 19:477**

**https://doi.org/10.1186/s12887-019-1859-y**

Following the publication of the article [1], the authors noticed that Fig. 3 used is not the updated version. The correct version is shown below.

**Fig. 3:**
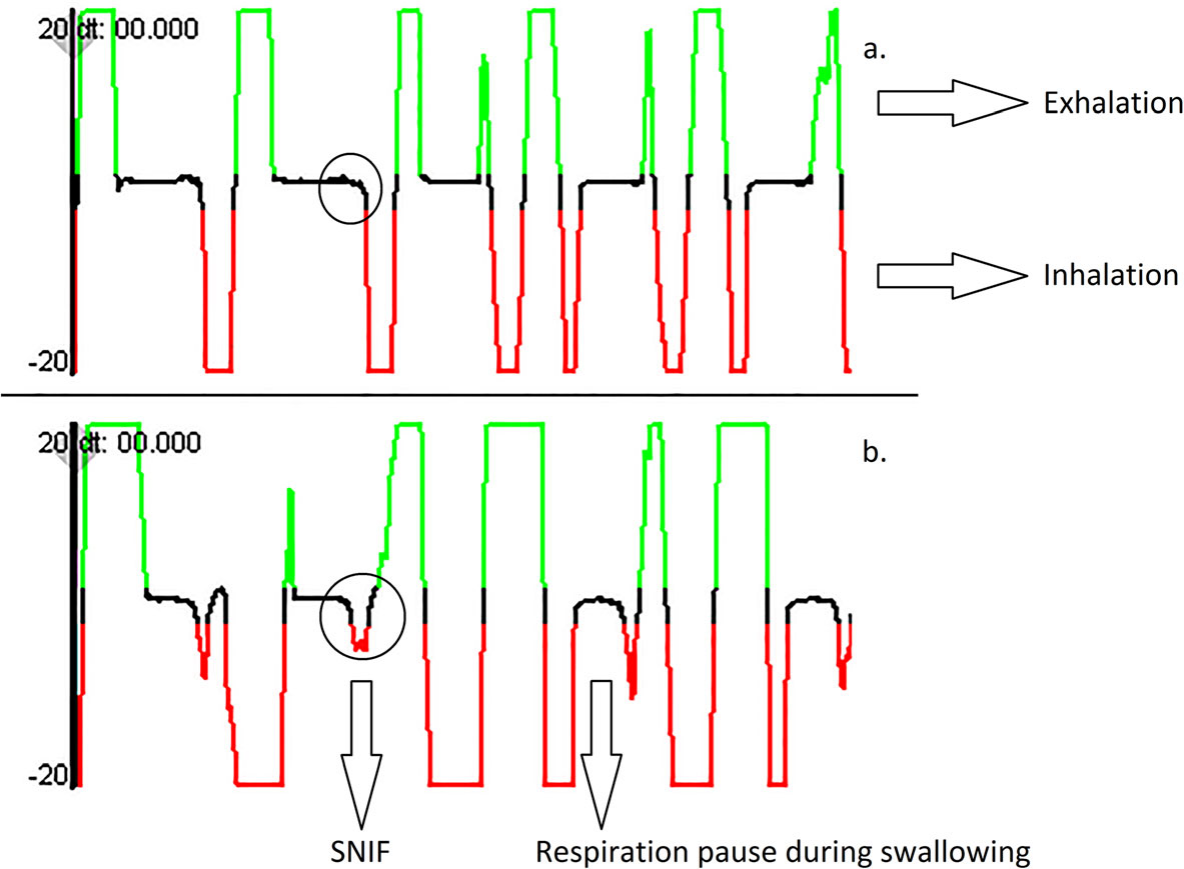
Nasal airflow measurement showing 7 s of nutritive sucking. Nasal airflow shows (**a**) no swallow non-inspiratory flow (SNIF), and (**b**) occurrence of SNIF during swallowing

The original article has been corrected.
